# Supplementary material for: Are carbon emissions trading and green financial instruments synergistic? -Comprehensive quantitative research based on content analysis
Source: PLoS One. 2024 Mar 7;19(3):e0298601. doi: 10.1371/journal.pone.0298601 (PMC10919601; doi:10.1371/journal.pone.0298601)
Supplement: S1 Data — (ZIP) [file pone.0298601.s001.zip › Data/地级市Z_F工作报告.zip!/╦─┤¿/╦∞─■╩╨/└┤╫╘╣1⁄2╓┌║┼í╛┬φ┐╦╩2╛▌═°í┐_2015.docx]

各位代表：

　　我代表市人民政府向大会报告过去一年的政府工作，对今年工作提出建议，请各位代表审查，并请市政协委员提出意见。

2014年工作回顾

　　过去一年，是全面深化改革的开局之年，也是近几年来经济发展十分困难的一年，经济下行的压力从没有减轻过。在市委的坚强领导下，在市人大、市政协的监督支持下，我们紧紧依靠全市人民，始终保持专注发展改革定力，正确把握发展大势，积极应对复杂局势，深入实施"六大兴市计划"，全市经济社会持续健康发展。

　　——经济运行稳中有进。全年实现地区生产总值809.6亿元，增长9.7%。其中：第一产业增加值139.2亿元，增长3.7%；第二产业增加值449.3亿元，增长11.4%，规模以上工业增加值增长12.0%，建筑业增加值增长10.8%；服务业增加值221.1亿元，增长9.7%。

　　——结构调整步伐加快。三次产业结构从上年的18.0：55.2：26.8调整为17.2：55.5：27.3。工业结构出现积极变化，电子信息产业产值增长17.2%，新材料产业产值增长59.2%，分别比全市工业平均水平高2.9个百分点、44.9个百分点。投资结构进一步优化，完成全社会固定资产投资913.7亿元，增长13.5%，民间投资占比达到67.2%。消费支撑作用明显增强，实现社会消费品零售总额348.2亿元，增长13.5%，消费对经济增长的贡献率达60%，提高5个百分点。

　　——人民生活持续改善。城镇居民人均可支配收入达到22790元，增长9.9%；农民人均纯收入达到9482元，增长11.6%。完成地方一般公共预算收入39.7亿元，同口径增长15.6%。在财政收支矛盾较大的情况下，坚持将财力向民生倾斜，民生支出达107亿元，占一般公共预算支出的68.5%。全市十大民生工程、21件民生实事全面完成。

　　2014年，困难比预料的多，结果比预想的好。我们统筹稳增长、调结构、促改革、惠民生等各方面工作，采取一系列既稳当前、又惠长远的举措，经济社会发展既有量的扩大，又有质的提升。

　　一、多措并举稳增长促后劲。在"三期叠加"的大背景下，去年一季度全市GDP增速下滑至2002年以来的最低水平，"稳增长、保目标"显得尤为迫切，并成为贯穿全年的工作主题。我们在年内相继出台了39条稳增长措施、9条促进房地产业平稳健康发展的意见、12条财税支持政策、加快发展服务业的意见、促进旅游业发展若干政策，以及八大成长型产业推进方案等，既稳定当期增长，又巩固增长预期，增强发展后劲。

　　狠抓企业稳增长。稳增长重点稳工业，稳工业重点稳企业。我们深入实施"大企业培育计划"，11户重点培育的工业企业完成主营业务收入318.5亿元，占规模以上工业企业的28.3%。积极落实创业就业和中小微企业帮扶政策，新增规模以上工业企业47户。下功夫解决企业在建设生产中遇到的土地、资金、能源等突出问题，创新推出"量子基金"贷款、"欣农贷"等金融产品，引导企业充分运用股市再融资、设备租赁、信托、债券等融资手段，实现资本市场融资66亿元，市内金融机构各项贷款余额增长23%。通过努力，工业用电量从3月起、银行存贷比从6月起逐月向好，规模以上工业30个行业大类中有27个行业保持增长。

　　狠抓投资稳增长。面对投资高位增长乏力的实际，坚持把稳投资作为稳增长的关键，一批夯基础、增后劲、利长远、惠民生的重大项目取得突破。328个"六大兴市计划"项目完成投资717亿元，实现年度计划的107%。机场迁建、遂广高速、遂西高速、中环线、观音湖隧道等重点项目有序推进。蓬溪220KV和金桥、仁里110KV输变电工程建成投运。全市人民期盼已久的武引蓬船灌区项目建议书获国家发改委正式批复，三仙湖水库加快建设，毗河供水一期、祥凤寨水库、群英水库如期开工。

　　狠抓消费稳增长。大力培育遂宁品牌，新增四川名牌产品13件、国家地理标志保护产品2件。积极参加"惠民购物全川行动""川货全国行"等省级市场拓展活动，精心组织川渝（遂宁）精品粮油展示展销会、第三届川渝汽车物流文化节、农产品博览会、五彩河东绿色生活博览会等展会，遂宁产品在市内外的影响力有效提升。启动"天虎云商"企业入网工程，235户企业通过电子商务平台有效拓展市场。积极开展"一刻钟便民服务圈"建设，新开办社区超市、便利店158家。

　　二、主动作为调结构促升级。针对阻碍发展的结构性问题，我们坚持重点突破、整体推进，产业结构、城乡结构、区域结构不断优化，县区经济竞相发展。

　　产业转型加快推进。我们在"加快赶"的同时"抓紧转"。突出抓创新驱动，市财政安排8200万元用于科技创新，新增国家级高新技术企业18家，省级以上创新型企业10家；引导传统企业加强技术改造，技改投资增长25.4%。推动高金食品与印纪传媒、华润锦华与创维数字完成战略重组。突出抓70个投资5000万元以上的重点工业项目，磨溪天然气净化厂二期、京石·海瑞克钻具成套装备等26个项目投产，江淮汽车西南基地、广义微电子6英寸芯片等44个项目加快推进。突出抓40个重点服务业项目，远成集团西部物流中心、遂宁软件外包产业园等8个项目纳入全省100个服务业重大项目，我市被确定为全省现代物流重点区域；五大旅游度假区建设有序推进，七彩明珠、观音湖生态湿地、中国红海成功创建国家4A级旅游景区，全市旅游收入增长20.4%。突出抓农业产业化，实现粮食总产155.6万吨，肉类总产32万吨；整合涉农财政资金11亿元集中用于5个现代农业园区建设，核心区初具规模；全市规范流转土地95万亩，新增农业产业化龙头企业13家。

　　城乡面貌不断改观。坚持把以人为核心的理念贯穿到城镇规划、建设、管理等各个环节，城镇化率提高1.5个百分点。城市总体规划获省政府批复，建立了规划督察员和乡村规划师制度，城乡规划空间管治制度基本形成。市城区大力实施玉龙路、南环路、开善东路改造和世界荷花博览园、公园路高架桥等54个环境提升项目，生态山水城、现代花园城、观音文化城加快建设。推进市域副中心建设和城镇集群发展，20个绿色重点小城镇、100个幸福美丽新村和5个新农村示范片加快建设，龙凤、沱牌等11个镇入选全国重点镇。深入开展"多城联创"，成为全国水生态文明城市建设试点市、省级节水型城市，被中国城市竞争力研究会评为"中国十佳宜居城市"。完成国控出川水质断面污染源综合整治项目，城市饮用水源水质达标率为100%。森林覆盖率提高0.54个百分点。

　　县区发展竞相突破。大力发展县域经济，底部基础进一步夯实。船山区以物流业为重点推动现代服务业集聚发展，物流港荣获"全国优秀物流园区"称号，中国物流论坛会址落户船山。安居区以江淮汽车为龙头的汽摩产业园初具规模，天然气产业园规划及项目引进有序推进。射洪县成为全省首批工业强县示范县、省级现代流通示范县和商标战略实施示范县，射洪经开区被列为省级知识产权试点园区，西部国际技术合作产业园取得积极进展。蓬溪县成为国家级现代农业示范区和全国首批依法治县工作先进县，成功举办首届西部门博会，金桥新区西部绿色食品饮料基地构架基本成型。大英县成为全国文化先进县、全国首批民生改善典型县和全省首批十大旅游强县，连续5年跻身中国最具投资潜力中小城市百强县。

　　三、坚定不移抓改革促开放。我们坚持以改革破除制约发展的体制机制束缚，以开放在更大范围内整合集聚资源，不断激活发展的内生动力和市场活力。

　　改革攻坚纵深推进。按照市委全面深化改革的"时间表"和"路线图"，完成120项重点改革任务。在简政放权方面，加大取消、调整和下放行政审批事项力度，市本级保留行政审批事项87项，成为全省行政审批项目最少的市州之一；大力推进工商登记制度改革，成为全省"先照后证"试点市，新登记设立公司1893户，增长63.5%。在国企改革方面，实施国企高管薪酬制度改革，49家市属国有企业整合为28家。在农村改革方面，加快推进农村产权制度改革，农村土地承包经营权确权登记工作在全省率先通过验收，全面完成农村集体建设用地使用权确权登记颁证和农村宅基地使用权确权登记，农村房屋确权登记办证率达82%，农村产权抵押贷款达1.08亿元。同时，深入推进城管体制改革，建立起"条块结合、以块为主"的市城区城管体制；深入推进投融资体制改革，包装推出21个总投资322亿元的鼓励民间投资示范性项目；稳步推进金融体制改革，大英信用联社成功改制为农商行。

　　开放合作成效明显。成功承办第三届中国（四川）国际物流博览会，精心组织2014绿色经济遂宁会议等特色活动，积极组织参加第十五届"西博会"，取得了主题营销和参展效果最好的成绩。大力实施招大引强，积极参加"中外知名企业四川行"等五大省级招商活动，主动"走出去"专题招商，全市到位市外资金420.6亿元，成功引进万达广场、卓达·蓝岛、中绿集团等投资10亿元以上项目24个。加快通关平台建设，遂宁海关开关运行，公用型保税仓库建成投运，告别了遂宁企业"外地报关"历史。全年进出口总额增长13.3%，实际利用外资增长9.1%。遂宁开发区成功创建省级外向型产业园区。继续深化"盟遂合作"、"农遂合作"、厅市合作，在项目、资金、人才等方面获得了更大支持。

　　四、千方百计惠民生促和谐。我们始终把改善民生作为一切工作的出发点和落脚点，兜住民生底线，推动社会事业发展，促进社会和谐稳定。

　　社会事业不断进步。成功承办省第十二届运动会、第八届残运会暨第三届特奥会，全民健身运动和群众体育活动蓬勃开展。新建公办幼儿园24所，义务教育保障经费和教育资助费用达5.1亿元，高考本科上线率达到50%，职业教育取得新突破。全面启动分级诊疗工作，县级公立医院全部实现药品零加成。稳步实施"单独两孩"政策。基本建成市县乡村四级公共文化服务体系，公共文化场馆全部实现免费开放。努力提升公交整体服务质量，完成市城区民营公交线路置换和公共自行车第三期建设工作，新增公交线路4条、公交车140辆。

　　社会保障持续加强。在全省率先建立统一的城乡居民基本养老保险和医疗保险制度，城乡居民大病保险试点工作取得明显成效。妥善解决被征地农转非人员养老保险历史遗留问题，全面启动实施新征地社保政策。加大重点人群保障力度，城乡低保对象月人均补差分别提高22元、11元，为2.7万名一、二级重度残疾人发放护理补贴2065万元，解决15.5万农村人口的饮水安全问题，6.3万农村贫困人口脱贫解困。连续三届被评为"中国城市公益慈善指数七星城市"。大力实施"百姓安居工程"，19110套保障性住房全部开工，建成17763套。建成通乡油路173.8公里、通村公路120.8公里、安全护栏472.1公里。积极稳定和扩大就业，城镇新增就业36797人，城镇登记失业率控制在4.15%以内。市城区应急备用水源工程投入使用，成为全省首个拥有双水源的城市。

　　社会大局和谐稳定。按照市委依法治市工作部署，积极推进法治遂宁建设。恢复设立市应急委，应急处突能力及管理水平逐步提升。健全安全生产监管责任体系，深化安全生产大检查，推进道路交通安全综合整治攻坚年等专项行动，安全生产保持平稳态势。全面提升反恐防暴等级，全市抢夺和抢劫案件分别下降10.1%、22.1%。健全食品药品安全"五级监管"体系，监管力量向源头和基层倾斜。大力解决信访突出问题，越级上访和集访人次持续下降。"12345"政府服务热线受理群众诉求48.3万件，办理满意率达到91%。国防动员、人防、民兵预备役建设、双拥、民族宗教、保密、档案、地方志和妇女儿童等工作扎实推进。

　　一年来，我们坚持把依法行政作为第一准则、高效执行作为第一标准、团结协作作为第一动力、清正廉洁作为第一品格，扎实推进政府自身建设。坚持常务会学法制度，强化行政决策合法性审查，加强行政决策风险评估，规范行政执法自由裁量权。加大政府购买公共服务力度，创新公共资源交易方式，得到省委、省政府的充分肯定。解决依法行政突出问题等工作成为全省典范。出台规范土地出让、工程项目招投标等重点领域"十项制度"，推进行政权力规范公开运行。整合卫生计生、食品药品等机构职能，稳步推进政府机构改革。深入开展党的群众路线教育实践活动，推进正风肃纪"9 6"突出问题集中整治，开展"庸懒散浮拖"问题专项整治，"三公"经费下降19.1%，政府发文数量下降62.2%，政府全市性会议减少45.5%。自觉把政府工作置于人大的法律监督、工作监督和政协的民主监督之下，办理人大代表建议139件、政协委员提案255件，回复满意率和基本满意率达99%以上。

　　各位代表，过去一年，面对经济下行压力持续加大的严峻形势，在市委的坚强领导下，我们把握住了经济工作大的取向，把握住了提振经济的关键环节，把握住了经济发展中的重大机遇，及时有效地化解和处理了发展中一系列危机和困难。全市上下的奋斗倍加艰辛，取得的成绩实属不易。这是省委、省政府和市委坚强领导的结果，是市人大、市政协监督支持的结果，是全市广大干部群众团结一心、拼搏实干的结果，是建市近30年来一届班子接着一届班子努力奋斗的结果。我代表市人民政府，向全市人民，向人大代表、政协委员，向各民主党派、工商联、无党派人士、各人民团体，向历届老领导和所有关心支持遂宁发展的各界朋友表示衷心感谢并致以崇高敬意！

　　各位代表，2014年《政府工作报告》明确的目标任务，是经市六届人大五次会议审议通过，是市人民政府向全市人民作出的庄严承诺。一年来，我们围绕目标任务，竭尽全力抓落实。但受国内经济整体放缓的影响，以及自身结构性矛盾的制约，地区生产总值、规模以上工业增加值、城乡居民收入与年初预期目标尚有差距，唐家渡电航工程、武引二期（射洪段）等项目建设没有达到时间进度要求。在此，我代表市人民政府向各位代表表示歉意，并郑重承诺，争取建设滞后的项目尽快赶上进度。

　　各位代表，总结去年的工作，我们清醒地看到，当前我市经济社会发展还存在诸多困难和问题。投资持续保持高位增长难度加大，新的消费热点不多，要素支撑乏力，经济向好发展的基础不牢固。服务业比重偏低，特别是工业中传统产业比重过大，部分重点企业生产经营困难，面临艰难的转产转型转向，加之国家从面上规范招商引资优惠政策，依靠传统的资源消耗引进增量调结构的空间受到制约，结构调整任务异常艰巨。房地产业不稳定，催生财政、金融等领域的潜在风险逐步显现。城乡群众住房难、上学难、看病难等问题还没有得到根本解决，保障和改善民生任务仍然很重。在政府系统中还不同程度存在"庸懒散浮拖"等不良现象，有的决策部署和工作措施还没落到实处，仍需进一步强化执行力建设。我们将以对党和人民高度负责的态度，正视问题，下深水研究对策措施，认真加以解决。

　　2015年主要任务

　　今年是全面完成"十二五"规划之年，也是遂宁建市30周年，发展改革任务艰巨繁重。在我们前进的道路上，追赶跨越和转型升级的"双重任务"特别艰巨，发展动力转换和内生动力不足的"双重课题"特别繁重，风险日益显现和防风险能力不足的"双重挑战"特别严峻。但我们仍然处在大有可为的重要战略机遇期，总体向好的发展基本面没有改变。特别是建市30年来，一届接一届打下的坚实基础、创造的良好条件、积蓄的强劲势能，将为经济增长提供强力支撑。我们要坚定信心，牢牢把握工作主动权，主动适应经济发展新常态，守住风险防控的底线，闯过转型攻坚的关口。

　　纵观发展大势，确定今年政府工作思路和目标，需要重点考虑三方面的因素。一是充分考虑新常态下的经济运行新形势。新常态不是一个经济周期，而是一个新时期、一个新时代，具有全面、深刻、持久的特征。新常态下，经济增速在较长时期内保持中高速的态势将不可逆转，经济结构转型升级的态势将不可逆转，房地产高位放缓对经济拉动作用减弱的态势将不可逆转，减少对土地、负债、牺牲环境等传统路径依赖的态势将不可逆转。在这样的大背景下，要求我们在稳增长、促改革、调结构、惠民生等多重目标中寻找平衡点，向改革创新要动力、要活力，既要稳住发展速度，又要转方式调结构，促进提质增效、可持续发展。二是充分考虑率先实现全面建成小康社会的目标需要。市委提出要在全省丘区率先实现全面建成小康社会，这是全市人民的共同愿望，也是政府推动发展的目标所在，我们一刻也不能懈怠。2014年全市人均GDP只占全省平均水平的69%左右。未来一段时期，坚持比全国全省快一些的发展速度，仍是我们的最强音。三是充分考虑增长动力转换的影响。从传统增长点转向新的增长点，这是发展进入新常态的重要特征。在新旧增长点的拉锯式转换中，经济短期波动性会显著增强，经济和社会领域的风险因素会叠加显现。我们必须对今年的困难有充分的估计，既要抓经济发展新动力的形成，又要抓综合风险的防控。应当坚持量力而行、尽力而为的原则，把握好发展的预期和节奏，合理确定目标任务。

　　今年政府工作总体要求是：深入贯彻党的十八大及十八届三中、四中全会和省委十届四次、五次全会，以及中央、省委经济工作会议精神，全面落实市委六届六次、七次全会和市委经济工作会议部署，主动适应经济发展新常态，坚持稳中求进工作总基调，深入实施统筹城乡、"三化"联动发展战略和"六大兴市计划"，加快转方式调结构促升级，着力防风险补短板增后劲，狠抓改革攻坚，推进依法治市，加强民生保障，突出创新创业，奋力实现经济持续健康发展和社会和谐稳定。

　　今年全市经济社会发展主要预期目标建议为：地区生产总值增长8.5%，全社会固定资产投资增长10.6%，社会消费品零售总额增长11.5%，地方一般公共预算收入增长10%，城镇居民人均可支配收入增长9.3%，农民人均纯收入增长11%。

　　一、转型升级是我们的必由之路，着力培优扶强，大力发展实体经济，打造产业竞争新优势

　　牢固树立"产业强则经济强"的理念，集中资源做强产业，夯实"稳"的基础，创造"进"的条件，抢占转型升级先机。

　　（一）推动工业转型升级。坚持一手抓成长型产业培育，一手抓传统产业改造，力争新增规模以上工业企业30户，规模以上工业增加值增长10%。大力培育天然气、机电与装备制造、新材料、电子信息等成长型产业，加快磨溪天然气净化厂三期、江淮汽车西南基地、卓达·蓝岛、创维数字、广义微电子6英寸芯片等63个投资5000万元以上的重点工业项目建设，打造一批新的增长点。推动食品饮料、石化等传统产业改造升级，支持沱牌舍得、美丰集团等企业进行战略重组，实施盛马化工重油深加工等技改项目。特别是抢抓纺织业回暖的机遇，巩固纺织行业规模优势，提升产业效益。创新政府抓工业的思路办法，设立产业发展基金，支持有发展前景的优势项目。加快实施园区"141"工程，完善园区配套功能，促进产业集群成链发展。

　　（二）加快提升服务业。优先发展现代物流、文化旅游、科技服务、养老健康等成长型服务业，稳步发展批零住餐等传统服务业，力争服务业增加值增长9.5%。加大扶持服务业发展政策措施落实力度，设立服务业发展引导资金，鼓励企业和个人创新创业。加快建设远成物流、铁路物流园等项目，支持生产加工企业主辅分离，推动物流业与三次产业深度融合。加快建设五大旅游度假区、创意文化产业园，深入开展"百A百星"创建行动，打造特色乡村旅游品牌，推动文旅融合发展。做实遂宁开发区高新技术创业服务中心，推进河东软件服务产业孵化中心等项目建设，加快建立专利代理、技术交易、成果孵化等服务体系。支持社会力量开办养老机构，加快建设遂宁?国际医疗健康产业园、螺湖国际养老社区等项目。

　　（三）稳步发展现代农业。稳定450万亩粮食播面，粮食总产达到156万吨以上。整合涉农资金，加快5个现代农业园区建设。大力发展优质粮油、优质果蔬、现代林业等产业，新建和改造提升粮经复合产业基地50万亩。实施畜牧业提质增效工程，推进国家现代畜牧业示范区和全省现代畜牧业深化试点市建设。加快农技推广和服务体系建设，强化农产品质量安全监管，打造遂宁农产品特色品牌。加强农业生产能力建设，完成11座病险水库整治，新建高标准农田10万亩，新增农村沼气用户7500户。创新农业经营体系，大力培育专业大户、家庭农场、农民合作社等新型经营主体，完善农民与企业利益联结机制，发展适度规模经营。

　　二、改革创新是我们的最大红利，着力动力转换，减少对传统路径的依赖，催生科学发展新动能

　　坚持把改革创新贯穿于经济社会发展各个领域，向改革要动力，以创新激活力，掀起大众创业、万众创新的热潮。

　　（一）深化重点领域改革。继续深化简政放权，把放管结合的改革推向深入，推进市县区联网审批。继续推进事业单位分类改革，逐步扩大事业单位法人治理结构建设试点范围。稳妥有序推进公务用车制度改革，创新公务出行保障方式。深化农村改革，全面完成农村土地承包经营权和农村房屋产权颁证工作，适时启动小型水利工程确权登记颁证工作，开展农村集体资产股份制改革试点。建立健全市、县区、乡镇三级农村产权交易平台。加快国资国企改革，完善现代企业制度，鼓励民间资本参与国企改制重组。深化财税改革，将政府的收入和支出全部纳入预算管理，清理规范税收等优惠政策，规范政府债务管理，做好"营改增"扩围工作。加快地方金融体系建设，推进城市商业银行改制上市，力争遂州农村信用联社改组为农村商业银行。推进不动产统一登记制度改革。

　　（二）大力实施创新驱动。实施"遂州英才招揽行动"，建立高新技术企业及人才落地支持政策，加快引进创新创业人才。引导企业与高校、科研院所联建"产学研"联盟，支持英创力、久大蓬莱盐化等企业组建研发中心。促进高新技术产业集群发展，支持物流港和射洪经开区申报省级高新技术产业园区，支持西部国际技术合作产业园打造全省一流的转型升级示范园区。市级应用技术研究与开发经费坚持"拨改投"，引导创新要素向企业流动，力争引进转化科技成果20项以上，新增国家高新技术企业8家以上。

　　（三）强化关键要素保障。加强银政企对接，支持银行创新金融产品，引导企业运用股市、债券等工具直接融资。规范民间投融资行为，引导民间资金合理流向实体经济。坚持招商项目用地评审和亩均税收制度，加大低效用地清理。实施城乡建设用地增减挂钩项目，力争增加周转指标1500亩。积极争取天然气指标地方留成和价格优惠，促进资源就地转化利用。加快遂西、乌木厅、窑湾110KV变电站等能源设施建设，提升能源供应稳定性。

　　三、扩大需求是我们的重要支撑，着力内需拉动，优化投资扩大消费，激发投资消费新活力

　　坚持从需求方面施策，从供给方面发力，优化投资结构，扩大消费需求，提升开放水平，使经济增长建立在内需持续扩大的稳固基础之上。

　　（一）保持投资合理增长。稳定政府性投资，重点投向必须保障的民生项目，以及既稳当前又利长远的基础设施项目。积极对接国省政策支持方向，争取重大水利、交通、能源、民生保障等领域的投资。积极运用政府与社会资本合作的PPP等投融资模式。大力实施"6382"项目投资计划，力争遂广和遂西高速遂宁段全面建成、凤台大桥和唐家渡电航工程开工、中环线完成主体工程，实施柳树电航枢纽区土建工程。加快推进"六大水利工程"，力争武引蓬船灌区、武引二期（射洪段）开工，三仙湖水库大坝主体工程完工，加快毗河供水一期、祥凤寨水库、群英水库建设。

　　（二）有效扩大消费需求。引导企业积极参加省级市场拓展活动，筹办好川渝（遂宁）精品粮油展示展销会、农产品博览会等促销活动，扩大遂宁产品市场占有率。顺应市场个性化、多样化的消费需求，以抓新产业、新产品、新网络促进消费，重点扩大家政、信息、旅游和教育文体等领域消费。创新推动电子商务发展，加快建设物流公共信息等7个电商平台，全力引进知名电商企业，建设区域性分拨中心、结算中心。实施"全企入网"工程，推动电子商务进农村，构筑城乡现代流通体系。结合养老保险并轨，完善机关事业单位工资制度，提高中低收入群众收入水平，进一步释放消费潜力。

　　（三）提高开放合作水平。积极融入"一带一路"和长江经济带等开放大格局，加强通关平台建设，加快综合保税区申建，启动国际陆港申建。大力培育外向型企业，进出口总额增长4%，实际利用外资到位6000万美元。加强国际友城建设，筹办好"侨资企业西部行"、"外企看四川—遂宁行"等活动。积极参与成都平原经济区协同发展，做好大英至三台、遂宁至中江、南（遂）潼高速公路和绵遂内铁路等项目前期工作。创新招商理念和方式，继续推进"两转变一并重"，主动适应招商新常态。重点围绕八大成长型产业，突出产业链招商、以商招商，力争引进投资10亿元以上项目15个。加强签约项目的跟踪督促，提高履约率、开工率、投产率。

　　四、城乡统筹是我们的最大潜力，着力新型城镇化，推进全域发展，构建竞相突破新格局

　　坚持全域发展，加强分类指导，推动县区特色化、差异化发展，形成整体推进、竞相突破的良好格局。

　　（一）加快推进新型城镇化。大力推进以人为核心的新型城镇化，力争城镇化率提高1.5个百分点以上。围绕 "绿色城镇·幸福生活"主题，加快推进绿色建筑试点城市、国家智慧城市试点等工作。加快市城区建设，重点推动河东二期、物流港、金桥、西宁片区等城市新区建设，实施滨江中路、渠河路改造等工程。支持射洪、大英、蓬溪县城发展壮大，加快建设市城区到各县区的快捷通道。深入实施"绿色城镇建设行动"，加快20个试点镇建设。开展"城市基础设施建设年行动"，加快城镇地下管网、公共服务设施等工程建设。加大城镇危旧房棚户区改造力度，积极推进货币化安置。加强改进城市管理，促进房地产市场平稳健康发展。

　　（二）统筹推进新农村建设。扎实抓好安居、蓬溪两个省级新农村示范县建设。实施"百村建设行动"，积极推行"申建制"等模式，加快100个幸福美丽新村建设和一批聚居点改造提升。加快"1 6"村级公共服务体系建设，打造一批传统文化村落，积极争创国家级和省级生态文明乡村。实施精准扶贫，加大对农村贫困地区的扶持力度，完成脱贫解困4.1万人。促进农村劳动力转移输出，提升劳务经济质效。落实好各项惠农政策，增加农民转移性收入。积极探索农民财产权实现形式，努力增加群众财产性收入。

　　（三）促进区域协调发展。建立完善县区协调发展的推进机制，避免同质化竞争。支持船山区围绕"一核五区"定位，发展物流、新材料、文化旅游、养老健康等产业；安居区围绕遂宁城市副中心、区域经济新引擎、都市农业新典范定位，发展机械装备、天然气、临港物流等产业；射洪县围绕全省工业强县示范县、县域经济排头兵、全面小康先行县定位，发展壮大食品、化工、纺织三大优势产业，扶持延伸新材料、机械制造、电子信息三大新兴产业；蓬溪县围绕国家现代农业示范区、全省产城新区示范县、西部特色文化旅游名县定位，发展现代农业、文化旅游等产业；大英县围绕新型工业县、优秀旅游城、生态示范区定位，发展壮大石化盐化、纺织机电、文化旅游等产业。加大沿成南高速城镇发展带、涪江沿岸城镇发展带城镇建设和产业发展力度，示范带动全市发展。

　　五、美丽遂宁是我们的共同家园，着力环境优化，提升地区软实力，打造宜居宜业新环境

　　环境就是竞争力。大力优化生态环境、政务环境、社会环境，用良好的环境赢取更多的发展机遇。

　　（一）营造优美的生态环境。深入实施新《环境保护法》，加强大气污染防治；加大饮用水源保护力度，实行水源地到水龙头全过程监管；严格实施环境准入制度，狠抓污染企业挂牌整治。深入推进城乡环境综合治理，开展农村生活垃圾专项治理行动，加快城镇污水垃圾处理设施建设。有序淘汰企业落后产能，完成节能减排目标。加强观音湖、螺湖等湿地公园建设，实施天然林保护、退耕还林后续工程。深入开展"多城联创"，力争成功创建国家环保模范城市，加快创建国家节水型城市、全国水生态文明城市，争创国家海绵城市。

　　（二）打造高效的政务环境。按照"五个一律"要求，全面清理行政审批前置条件，逐步取消非行政许可审批事项，清理规范与行政审批有关的中介服务。完善政务服务体系，坚持导办员、重大项目VIP室等机制，方便群众和企业办事。严格按照公共资源交易目录，将政府购买中介服务、特许经营权等纳入交易平台，积极探索电子竞价等机制，确保公共资源交易规范运行。继续办好"12345"政府服务热线。

　　（三）创建平安的社会环境。坚持以法治为引领，创新社会治理方式。善于用法治思维和法治方式化解社会矛盾，解决好土地征用、房屋拆迁、企业改制等引发的矛盾。深入开展"法律七进"活动，引导群众自觉守法、遇事找法、解决问题靠法。坚持重大决策社会稳定风险评估，完善"大调解"工作体系，推动"诉访分离"。严厉打击"两抢一盗"，提升街面见警率，织密社会治安防控网。严厉打击非法集资行为，保障群众财产安全。严厉打击食品药品领域不法行为，确保群众"舌尖上的安全"。深化道路交通、建筑施工、危化物品等领域综合整治，推进隐患排查治理常态化，保持安全生产平稳态势。

　　六、改善民生是我们的从政之责，着力民生优先，回应人民美好期待，不断开创民生事业新局面

　　坚持从群众需求角度出发，切实解决群众最需、最盼、最急的问题，不断满足群众对美好生活的新期待。

　　（一）倾力办好民生大事。坚持保基本、兜底线、促公平，多做雪中送炭的实事好事，确保民生支出占一般公共预算支出的比重达到69%。继续实施好十大民生工程，突出办好21件民生大事：改善农村通村公路400公里；解决17.44万农村人口及学校师生安全饮水问题；改造城市危旧房棚户区14463套；改造农村危旧房1827户；向农民工定向出租240套公租房；为103080名老人提供居家养老服务；提高60岁以上城乡居民基础养老金发放水平；帮助1100名在校大学生和高校毕业生创业就业；继续提高城乡居民医疗报销水平；为"老幼病孕"等提供免费的基本公共卫生服务；为300名贫困白内障患者实施复明手术；为边远贫困地区农村教师建设60套周转房；提高19所公办普通高中办学经费；免除3600名民办中职学校三年级学生学费；治理5处地质灾害隐患点，避险搬迁安置87户农户；对所有村级公共服务设施运维提供补助；建立3个县级残疾人服务中心；全面推进"城市一卡通"遂州通工程；为公办学校配备安保人员；为80岁以上高龄老人发放补贴；免除婚姻登记证书工本费。

　　（二）协调发展社会事业。探索教育集团化办学，推进教师"县管校聘"改革试点。积极发展学前教育，新建、改扩建16所公办幼儿园。大力改善义务教育薄弱学校基本办学条件，促进城乡义务教育均衡发展。推进科教园区建设，力争开工建设1所大学。加快推进市中心医院河东院区二期、市妇女儿童医院、市民康医院迁建等项目建设。加强基层卫生力量，探索推进"县招乡用"、"乡招村用"试点。稳步推进分级诊疗工作。开工建设市文化馆、图书馆、博物馆新馆，筹办好"第三届涪江文化艺术节"、"中国观音文化旅游节"。保持体育场馆等设施公益性质，提高利用率。承办好省第八届老年人运动会。加快数字档案馆建设。人口自然增长率控制在4‰以内。

　　（三）完善社会保障体系。抓好城镇就业困难人员等群体就业工作，城镇登记失业率控制在4.2%以内。提高城乡居民医保补助标准，扩大社保覆盖面。全面落实新征地社保政策，努力实现新征地农民应保尽保。完善社会救助体系，城乡低保累计月人均补助水平分别达到230元、110元。加快市残疾人康复中心建设，深化残疾人"量服"工作。实施农村公路攻坚行动，着力解决"最后一公里"问题。积极培育和规范发展社会服务组织，继续加大政府购买公共服务力度。强化国防动员和后备力量建设，做好人防、双拥、民族宗教等工作。精心编制经济社会发展"十三五"规划。

　　加快建设法治政府

　　2015年，我们将按照"三严三实"标准，围绕建设法治遂宁，加快建设职能科学、权责法定、执法严明、公开公正、廉洁高效、守法诚信的法治政府。

　　一、坚持依法行政，习惯在法治的轨道上用权

　　依法履职用好权。坚持用制度规范权力，推进政府机构、职能、权限、程序、责任法定化，推行政府权力清单、责任清单、负面清单三大清单制度。严格执行"十项制度"，规范行政权力运行，消除权力设租寻租空间。解决一批群众反映强烈的依法行政中的具体问题。

　　科学决策行好责。坚持把合法性审查作为行政决策的第一要件，把部门论证、公众参与、成本效益分析、集体讨论决策等作为重大决策的必经程序。建立行政决策终身责任追究制及责任倒查机制。坚持政府常务会学法制度，推动政府部门办公会议学法，完善政府法律顾问制度。

　　规范公正执好法。减少市县执法队伍种类，在食品药品、农业、安全生产、城乡建设等领域推行综合执法。完善行政执法程序，纵深规范行政执法自由裁量权，挤压执法"弹性"空间。探索建立行政执法争议快速处理机制。健全行政执法与刑事司法衔接机制，实现行政处罚和刑事处罚无缝对接。

　　二、坚持阳光理政，习惯在监督的环境里工作

　　加强权力监督。自觉接受市人大及其常委会的法律监督、工作监督和市政协的民主监督，虚心听取各民主党派、工商联、无党派人士、各人民团体的意见建议。坚持以公开为常态、不公开为例外，推进财政预算、重大建设项目、重大决策部署等信息公开。加强监察、审计等专门监督，主动接受媒体监督。

　　完善绩效管理。根据县区、园区实际，量体裁衣制定考核指标，加大民生、财政收入等指标权重。建立纵向到底、横向到边的责任体系，完善差异化考核办法。拉开考核等次及奖励差距，鲜明以实绩论英雄的考核导向。

　　强化效能建设。坚持权责对等，实行层级管理、分线负责，把重大工作具体化、项目化、责任化。充分发挥县区和部门敢于担当、奋勇争先的主观能动性，做到在省级层面有声音、在市州有影响、在群众中有威信。构建大督查机制，打通抓落实的"最后一公里"，力破"中梗阻"。

　　三、坚持勤廉从政，习惯在清新的风气中生活

　　常怀为民之心。时刻把群众的冷暖安危放在心上，坚持为民着想、为民解难、为民造福，把权力用在为人民谋利益上。顺应群众期待，群众最盼望解决什么问题，就抓紧解决什么问题。

　　弘扬务实之风。坚持以政府的"勤奋指数"提升遂宁的"发展指数"，以干部的"辛苦指数"提升群众的"幸福指数"。树立功成不必在我的理念，发扬踏石留印、抓铁有痕的精神，到一线、进现场、解难题，多做打基础利长远的实事。坚持精简会议和文件，做到文不过夜、事不隔天、会不开长。

　　筑牢清廉之堤。严格落实党风廉政建设责任制，健全廉政风险防控体系。坚决纠正损害群众利益的不正之风，严厉查处各类违法违纪案件。巩固党的群众路线教育实践活动成果，主动适应政府工作新常态，严控"三公经费"，自觉过紧日子，让群众过好日子。

　　各位代表，新常态下，政府责任更加重大，路途更加艰辛，使命更加光荣。让我们在省委、省政府和市委的坚强领导下，同心同德、克难攻坚、砥砺奋进，为建设大而美、富而强、优而雅的新遂宁而努力奋斗！
